# Ultrasound Characterization of Patellar Tendon in Non-Elite Sport Players with Painful Patellar Tendinopathy: Absolute Values or Relative Ratios? A Pilot Study

**DOI:** 10.3390/diagnostics10110882

**Published:** 2020-10-29

**Authors:** José L. Arias-Buría, César Fernández-de-las-Peñas, Jorge Rodríguez-Jiménez, Gustavo Plaza-Manzano, Joshua A. Cleland, Gracia M. Gallego-Sendarrubias, Ibai López-de-Uralde-Villanueva

**Affiliations:** 1 Department of Physical Therapy, Occupational Therapy, Rehabilitation and Physical Medicine, Universidad Rey Juan Carlos, 28922 Alcorcón, Spain; joseluis.arias@urjc.es (J.L.A.-B.); jorge.rodriguez@urjc.es (J.R.-J.); 2 Cátedra Institucional en Docencia, Clínica e Investigación en Fisioterapia: Terapia Manual, Punción Seca y Ejercicio Terapéutico, Universidad Rey Juan Carlos, 28922 Madrid, Spain; 3 Department of Radiology, Rehabilitation and Physiotherapy, Universidad Complutense de Madrid, 28040 Madrid, Spain; gusplaza@ucm.es (G.P.-M.); ibailope@ucm.es (I.L.-d.-U.-V.); 4 Instituto de Investigación Sanitaria, Hospital Clínico San Carlos, 28040 Madrid, Spain; 5 Department of Public Health and Community Medicine, Tufts University School of Medicine, Boston, MA 02155, USA; joshua.cleland@tufts.edu; 6 Department of Physical Therapy, Universidad Camilo José Cela, Villanueva de la Cañada, 28692 Madrid, Spain; gmgallego@ucjc.edu

**Keywords:** patellar tendinopathy, ultrasound, cross-sectional area, thickness, ratios

## Abstract

Imaging findings in patellar tendinopathy are questioned. The aim of this pilot study was to characterize ultrasound measures, by calculating ultrasound ratio and neovascularization of the patellar tendon in non-elite sport players with unilateral painful patellar tendinopathy. Cross-sectional area (CSA), width, and thickness of the patellar tendon were assessed bilaterally in 20 non-elite sport-players with unilateral painful patellar tendinopathy and 20 asymptomatic controls by a blinded assessor. Ultrasound ratios were calculated to discriminate between symptomatic and asymptomatic knees. The Ohberg score was used for characterizing neovascularization. We found that non-elite sport players with patellar tendinopathy exhibited bilateral increases in CSA, width, and thickness of the patellar tendon compared to asymptomatic controls (Cohen d > 2). The ability of ultrasound ratios to discriminate between painful and non-painful patellar tendons was excellent (receiver operating characteristic, ROC > 0.9). The best diagnostic value (sensitivity: 100% and specificity: 95%) was observed when a width ratio ≥ 1.29 between the symptomatic and asymptomatic patellar tendon was used as a cut-off. Further, neovascularization was also observed in 70% of non-elite sport players with unilateral patellar tendinopathy. A greater CSA ratio was associated with more related-disability and higher tendon neovascularization. This study reported that non-elite sport players with painful unilateral patellar tendinopathy showed structural ultrasound changes in the patellar tendon when compared with asymptomatic controls. Ultrasound ratios were able to discriminate between symptomatic and asymptomatic knees. Current results suggest that ultrasound ratios could be a useful imaging outcome for identifying changes in the patellar tendon in sport players with unilateral patellar tendinopathy.

## 1. Introduction

Tendinopathy can occur in tendons that receive excessive loads and can result in considerable pain and disability [1]. While some tendinopathies can occur in the middle portion of the tendon, e.g., Achilles, others occur in bone insertion sites, e.g., patella. The prevalence of patellar tendinopathy (jumper’s knee) can reach 50% in people practicing athletic activities requiring jumping or landing [2]. Lian et al. examined the prevalence of patellar tendinopathy in 613 elite athletes from different sports and reported an overall prevalence of 14% across all the sports; however, prevalence data varied considerably depending on the sport, ranging from 0% in cycling to 40% in male volleyball players [3]. A more recent study found that 25% of Australian football players suffered from patellar tendon problems during the season [4]. In addition, patellar tendinopathy is also present in non-elite sport players with an overall prevalence of 8.5% [5].

Tendons that are exposed to considerable loading and releasing of stored energy repetitively can potentially develop tendinopathy [1]. If the injury is acute, inflammation occurs, the stress on the tendon is dramatically reduced, and, therefore, healing can potentially occur quickly. However, this process does not happen in those cases where repetitive loading and releasing of energy occurs such as in the typical population experiencing patellar tendinopathy where the tendon begins to degrade, resulting in a tendinopathy. Unfortunately, the pathophysiology of patellar tendinopathy is not properly understood and seems to be multifactorial. A number of plausible theories have been proposed over the past few decades. The first theory includes the presence of collagen degradation/disorganization of the cellular matrix which is not able to adjust to the demands placed upon the tendon [1,6]. The presence of neovascularization has also been proposed as another mechanism, as it is considered a sign of persisting hypoxia and failed tendon repair attempts [7]. In fact, the presence of neovascularization in abnormal patellar tendons has been associated with higher levels of pain and lower functional scores in elite active volleyball players [8].

It has been found that structural changes observed on ultrasound imaging can be associated with an increased risk of developing symptoms [9]; nevertheless, tendon pain is not always associated with the presence of abnormal findings on imaging studies [10]. In fact, there is a limited relationship between tendon imaging pathology and presence of symptoms since structural tendon changes can exist in the absence of symptoms, and symptoms can be present without the presence of imaging changes [11]. However, ultrasound imaging continues to be used as a confirmatory tool for a diagnosis of patellar tendinopathy. In fact, several studies have reported the presence of tendon imaging changes and/or neovascularization in the patellar tendon in elite sport players, both junior (volleyball players [12] or basketball players [13]) and adults (professional volleyball players [14] or basketball players [15]) The systematic review conducted by Obst et al. found inconsistent evidence supporting the presence of imaging changes in the patellar tendon in individuals with patellar tendinopathy [16].

Few studies have investigated the presence of patellar tendinopathy in non-elite sport-player populations. Additionally, most studies have investigated absolute gross measures of the tendon, e.g., thickness or cross-sectional area, but not potential ratios between the ultrasound measures. In fact, Obst et al. recommended that future studies should compare morphological changes of the patellar tendon between symptomatic and the contra-lateral asymptomatic extremity, but also with matched healthy controls [16].

Therefore, the aims of this pilot study were: (1) to describe the differences in gross ultrasound measures of the patellar tendon between non-elite sport players with unilateral patellar tendinopathy and asymptomatic healthy controls; (2) to characterize ultrasound measure ratios of the patellar tendon between symptomatic/asymptomatic knees to discriminate unilateral patellar tendinopathy; (3) to determine differences in those gross ultrasound measures depending on the presence or absence of neovascularization in the patellar tendon in non-elite sport players with unilateral painful patellar tendinopathy; and (4) to determine the possible association between symptoms with gross ultrasound measures, ultrasound ratios and the presence of neovascularization in the patellar tendon in non-elite sport players with unilateral patellar tendinopathy.

Our hypotheses were: (1) non-elite sport players with unilateral patellar tendinopathy will exhibit higher ultrasound gross measures in the patellar tendon than asymptomatic subjects; (2) ultrasound ratios will discriminate between painful and non-painful knees in individuals with painful patellar tendinopathy; (3) the presence of tendon neovascularization is associated with worse gross ultrasound imaging measures and ratios; and 4, ultrasound ratios and tendon neovascularization, but not gross ultrasound measures, will be associated with clinical pain symptoms in non-elite sport players.

## 2. Methods

### 2.1. Participants

**Figure 1:**
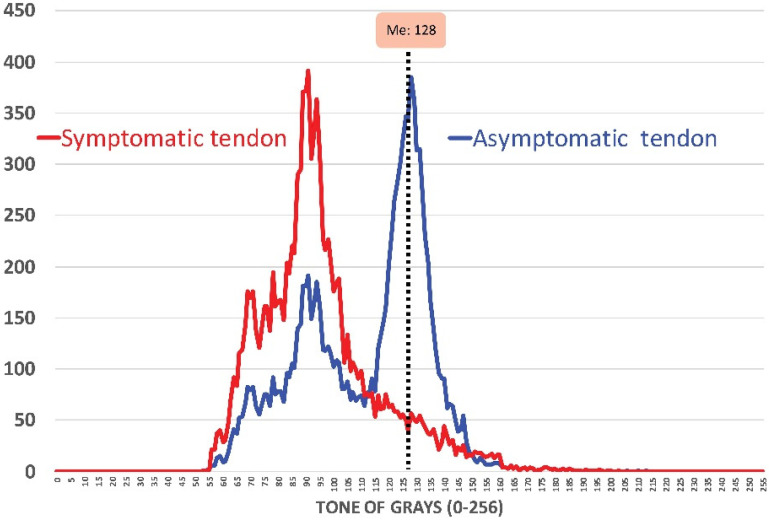
Tendon histogram (tone of grays) confirming the presence of hypoechoic areas in the symptomatic versus the asymptomatic tendon in non-elite sport players. Me: Median value.

It has been reported that patellar tendon pain is more prevalent in males than in females [1,2,3,4,5]. Further, patellar tendon structure is gender-dependent, since males had significantly greater non-uniformity than females [17]. Similarly, Ağladıoğlu et al. described differences in the patellar tendon between smokers and non-smokers [18]. Therefore, we included male, non-smoker, non-elite sport players with patellar tendinopathy to avoid the influence of these variables. Twenty (*n* = 20) non-elite sport players with a diagnosis of unilateral painful patellar tendinopathy referred to a physical therapy clinic by their sports physician were recruited between January 2019 and January 2020. To be eligible to participate, participants had to be diagnosed with painful patellar tendinopathy based on both clinical and imaging findings as follows: (1) pain located at the patellar pole associated with physical activity involving increased load in the tendon; (2) tenderness/pain to palpation on the patellar pole; and, (3) presence of a hypoechoic area in the tendon substance on greyscale ultrasound image. Figure 1 graphs the histogram confirming the presence of hypoechoic areas in symptomatic tendons in non-elite sport players.

Exclusion criteria included: (1) bilateral knee pain symptoms; (2) previous history of surgery; (3) having received physical therapy in the last six months; (4) taking regularly medication; or (5) professional/elite sport players. Further, 20 asymptomatic non-elite sport players with no history of knee pain symptoms in the previous two years and no presence of hypoechoic areas in the tendon substance were also recruited. The study was approved by the Ethical Local Committee of Universidad Rey Juan Carlos (URJC 2018-33) and was conducted following the Helsinki Declaration. All participants provided informed consent prior to their participation.

### 2.2. Pain and Related-Disability

Demographic data including age, weight, height, history of tendinopathy, duration of symptoms, medication and comorbidities were collected during a clinical interview [19]. A 10-point Numerical Pain Rating Scale [20] (NPRS; 0: no pain, 10: maximum pain) was used to assess the mean and the worst level of knee pain experienced the preceding week during their sports activity. The mean was used in the statistical analysis. Related-disability was assessed with the Victorian Institute of Sport Assessment (VISA-P) score [21]. This scale consists of eight items, six evaluating pain during daily activities and functional tests on a 10-points numeric pain rate scale, and two items providing information on sports participation (categorical response options). The VISA-P total score ranges from 0 to 100 points with higher scores representing lower related-disability. It has been suggested that 80 points in the VISA-P score is a potential determinant of patellar tendinopathy [21]. In this study the Spanish version of the VISA-P was used, which has been shown to be valid, reliable, sensitive to clinical changes and comparable to the original version [22].

### 2.3. Ultrasound Imaging of the Patellar Tendon

Ultrasound images were obtained with Shenzhen Mindray Co, M7 model, Nanshan, Shenzhen, China, ultrasound equipment and with a 10-MHz linear array transducer in grey scale B-mode by an assessor with ten years of experience in musculoskeletal ultrasound assessment and who was blinded to the subject’s condition. The depth was adjusted at 3 cm and the focal length was adjusted to the inferior paratenon. All measurements were assessed following the guidelines from the European Society of Musculoskeletal Radiology [23].

For the imaging, participants were lying supine with both knees flexed at 30°. A pillow was placed under the popliteal space during the examination and knee flexion was monitored with an inclinometer. This knee position avoids possible anisotropy related to the concave profile as a result of posterior thigh muscles and knee extension. The linear transducer was placed centrally on the patellar tendon in a longitudinal direction. Cross sectional area (CSA) and width (lateral dimension) were assessed on a transverse (short-axis) view whereas thickness (superior to inferior dimension) was assessed on a longitudinal (long-axis) view over this point. This procedure has obtained good to excellent reliability (ICC 0.70–0.95) for assessing patellar tendon thickness [24,25]. Further, assessment of the CSA of the patellar tendon has also shown to be as reliable as thickness assessment, showing high level of intra-observer compliance [26]. In our study, the mean of two measurements was considered in the analysis as recommended by Skou & Aalkjaer [24]. All assessments were taken at a location placed at 5 mm inferior to the apex of the patella since this point is the area of symptoms most reported by patients with patellar tendinopathy and has been used in previous studies [26].

**Figure 2:**
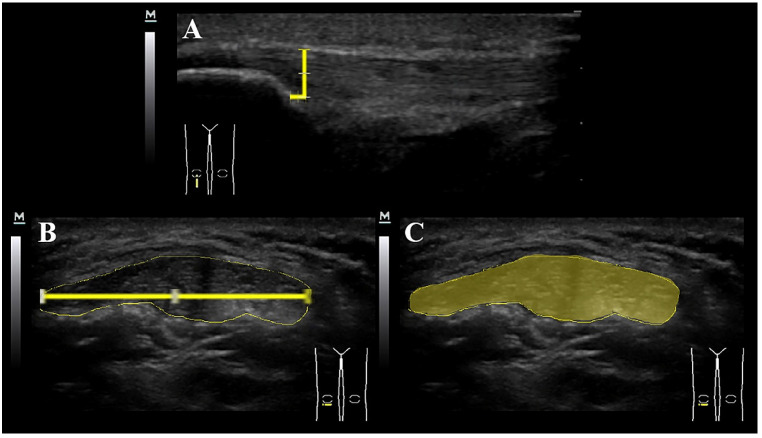
Ultrasound assessment of patellar tendon thickness (**A**), width (**B**) and cross-sectional area (**C**).

Once the images were captured, they were transferred to offline ImageJ 1.8^®^ Software for calculating thickness, width and CSA of the patellar tendon (to the nearest 0.01 mm). The tendon borders were defined inferiorly as the first hyperechoic region between the subcutaneous tissue and the deep fascia layer (Figure 2).

### 2.4. Neovascularization and Doppler Assessment of the Patellar Tendon

In our study, the Doppler insonation was 6.6 MHz. For the color parameters, a low-wall filter (WF191) and a low-speed scale (pulse repetition frequency, PRF 1.2 KHz, 0.5 KHz–14.8 KHz) were selected. Nyquist Frequency referred to the folding frequency of a sampling system that was maintained on a scale of −6.8 to 8.8 cm/s. The color gain was maintained in G53 at a dynamic range, and in general, 30 to 160 dB were used. The size of the color box (region of interest, ROI) was 3 cm^2^ (2 cm × 1.5 cm).

**Figure 3:**
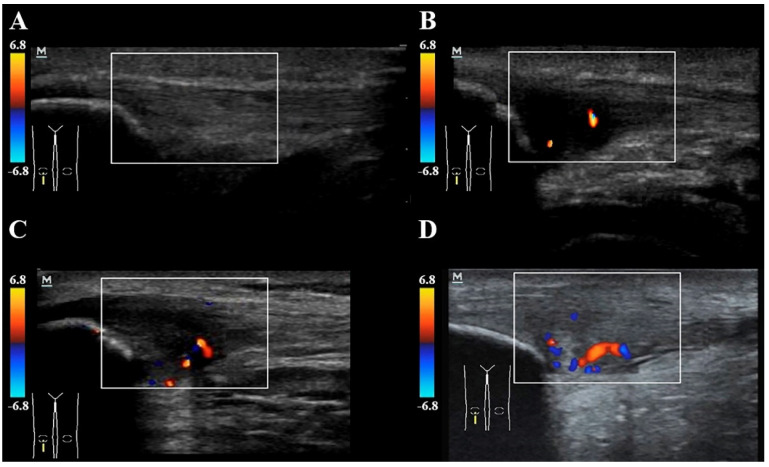
Intra-tendinous Doppler analysis for identifying neovascularization (**A**) grade 0: no visible vessels; (**B**) grade 1: 1 to 2 vessels within the ROI; (**C**) grade 2: 3 to 5 vessels within the ROI; (**D**) grade 3: vessels in up to 30% of the ROI. ROI: Region of Interest.

For the Doppler assessment, the knee was extended to relax the quadriceps muscle in order to prevent physical constriction of the blood vessels. The pressure of the probe was also kept to a minimum to avoid obliteration of small vessels. The number of micro-vessels was calculated in both groups by a second assessor with 15 years of experience in US musculoskeletal assessment and who was also blinded to the subject’s condition. The mean of two measurements was used in the analysis. Intra-tendinous power Doppler flow (neovascularization) was graded as follows: grade 0 (no vessels visible); grade 1 (1 to 2 vessels within the ROI); grade 2 (3 to 5 vessels within the ROI); grade 3 (vessels in up to 30% of the ROI); grade 4 (vessels in 30–50% of the ROI); or grade 5 (vessels in >50% of the ROI) (Figure 3). This neovascularization score is a modification of the system previously described [27,28] and used in the patellar tendon [29].

### 2.5. Statistical Analysis

Data were analyzed with the SPSS version 21.0 (SPSS Inc., Chicago, IL, USA). The normal distribution of the data was assessed with the Shapiro-Wilk. Descriptive data were presented as means (standard deviations, SD) for continuous and *n* (%) for categorical variables, respectively. No side-to-side differences were found in the asymptomatic control group; therefore, the mean value of both sides was used in the main analysis.

Separate 2-tailed paired *t*-tests were used to determine the differences in thickness, width and CSA in non-elite sport players with patellar tendinopathy between their symptomatic and asymptomatic tendons. In addition, separate paired *t*-tests were used to determine the differences in thickness, width and CSA between non-elite sport players with patellar tendinopathy (symptomatic and asymptomatic tendons, separately) and controls (mean value). Effect sizes (Cohen’s d) were classified as small (0.20–0.49), medium (0.50–0.79) or large (≥0.8) [30] and calculated for each post hoc comparison as follows:

(1) $$Cohen’s\;d_{non-paired\;comparisons}=\frac{\left|mean_{A}-\;mean_{B}\right|}{\sqrt{\frac{s_{A}{}^{2}+s_{B}{}^{2}}{2}}}$$

(2) $$Cohen’s\;d_{paired\;comparisons}=\frac{\left|mean_{A}-\;mean_{B}\right|}{SD_{\left|m_{A}-\;m_{B}\right|}}$$

*S_A_*: Standard deviation group A; *S_B_*: Standard deviation group B; *SD*: Standard deviation. 

To evaluate the validity of ultrasound measures to discriminate between symptomatic and non-symptomatic knees in individuals with patellar tendinopathy, the following ratios were calculated: (1) thickness ratio: symptomatic tendon thickness/asymptomatic tendon thickness; (2) width ratio: symptomatic tendon width/asymptomatic tendon width; and, (3) CSA ratio: symptomatic tendon CSA/asymptomatic tendon CSA. The creation of these ratios allowed us to quantify the relationship between morphology of the symptomatic and asymptomatic tendons in individuals with patellar tendinopathy. In such a scenario, the ratios are not affected by the within-individuals variability. The ability of ultrasound measurement ratios to detect painful knees in patients with painful patellar tendinopathy was evaluated by using the area under the receiver operating characteristic (ROC) curve, considering it values as excellent discrimination (≥0.9); moderate discrimination (≥0.7); or poor discrimination (<0.7) [31]. For each ratio, the optimal cut-off point was determined using the Youden index. Sensitivity, specificity, positive predictive value (PPV), negative predictive value (NPV), positive likelihood ratio (LR), and negative LR were calculated. Validity was considered acceptable when at least 70% sensitivity and 50% specificity were obtained [32]. Similarly, the same analysis was also conducted with ultrasound gross measures, i.e., CSA, width, or thickness (Supplementary Table S1).

The non-parametric Mann–Whitney U Test was used to determine the differences in clinical features (i.e., time with pain, pain intensity, VISA-P) and ultrasound measures (i.e., thickness, width and CSA gross values and ratios) depending on the presence (grades I–V) or the absence (grade 0) of neovascularization. Finally, the Spearman correlation rho test (95% CI) was applied to assess the associations between clinical features and ultrasound outcomes. In general, *p*-values < 0.05 are set as statistically significant; however, due to the inclusion of three main ultrasound imaging comparisons, the Bonferroni correction was applied (corrected significance level *p* < 0.05/3 < 0.017).

## 3. Results

Twenty male non-elite sport players with unilateral painful patellar tendinopathy (mean age: 45, SD: 4.5 years) and 20 asymptomatic non-elite sport players (mean age: 42.5, SD: 5.5 years) acting as controls were included. Anthropometry and clinical characteristics of the subjects are observed within Table 1. Sport players with patellar tendinopathy exhibited lower height and higher BMI than controls.

### 3.1. Association between Gross Ultrasound Measurements and Pain and Related-Disability

No significant linear association between ultrasound gross measures, i.e., width, thickness or CSA, and clinical features, i.e., time with pain, pain intensity and related-disability, was observed (Table 2).

### 3.2. Between-Groups Ultrasound Measurements of the Patellar Tendon

Non-elite sport players with patellar tendinopathy exhibited bilateral increases in all dimensions of the patellar tendon when compared to controls (all, *p* < 0.001). Additionally, the painful tendon had a significant increase in thickness, width and CSA compared to the non-painful tendon in those with patellar tendinopathy (all *p* < 0.001). Between-groups differences were large (d > 2) in all comparisons. Between-groups multiple comparison can be observed in Table 3.

### 3.3. Discriminant Validity of Ultrasound Measurement Ratios

The ability of ultrasound ratios (thickness, width or CSA) to discriminate the presence of the painful knee was excellent, with an area under the ROC curve greater than 0.9 in all ratios. The optimal cut-off points proposed for each ratio showed Youden indexes ≥0.85 and presented a +LR ≥ 6.7 and a −LR ≤ 0.06, so their discriminate validity was adequate. The best diagnostic validity value (sensitivity: 95% specificity: 100%) was found when a value ≥1.47 in the width ratio between the symptomatic and asymptomatic patellar tendons was used as a cut-off point. Table 4 shows the validity of ultrasound ratios for discrimination of painful versus non-painful knees in non-elite sport players with unilateral patellar tendinopathy.

### 3.4. Association between Ultrasound Ratios and Pain and Related-Disability

**Figure 4:**
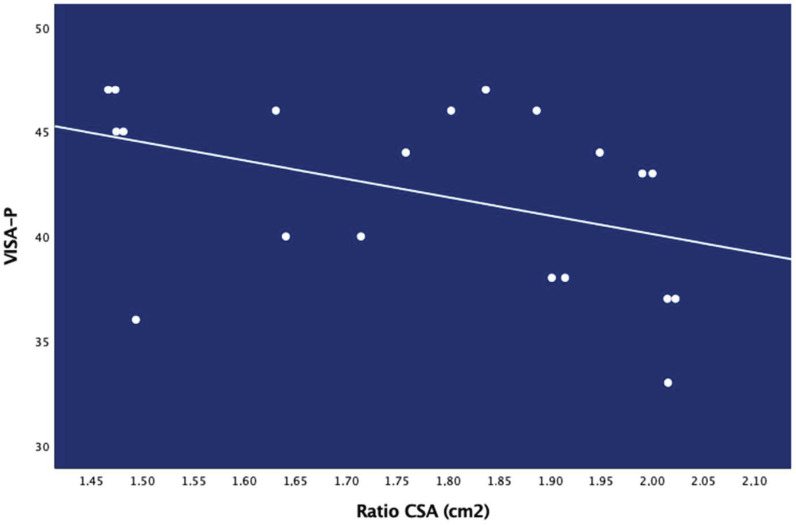
Negative linear association between Cross-Sectional Area (CSA) ratio and related-disability (VISA-P, Victorian Institute of Sport Assessment-Patella score).

No significant association between ultrasound ratios, i.e., width, thickness or CSA ratios, and clinical features, i.e., time with pain, pain intensity and related-disability was observed, except for a negative association between CSA ratio and VISA-P score (rs: −0.581; *p* = 0.007, Figure 4): the greater the CSA, the lower the VISA-P score (the more related-disability) (Table 5).

### 3.5. Clinical and Ultrasound Measurements by Neovascularization

Neovascularization was present only in the painful knee of non-elite sport players with patellar tendinopathy. No significant differences in pain intensity and related-disability depending on the presence or not of tendon neovascularization were found (all, *p* > 0.184). Similarly, ultrasound imaging measures (i.e., thickness, width, CSA) were not significantly different according to the presence or absence of neovascularization (all, *p* > 0.276). Only the CSA ratio was significantly (*p* = 0.017) different between non-elite sport players without neovascularization (grade 0) and those with neovascularization (grade I–II): non-elite sport players with tendon neovascularization exhibited higher CSA ratio than those without neovascularization (Table 6).

**Figure 5:**
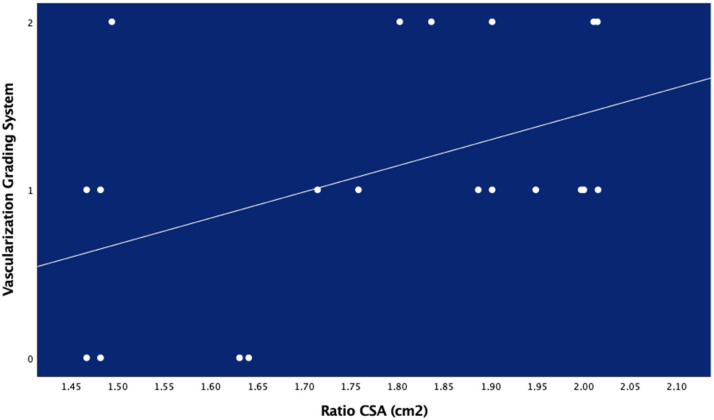
Positive linear association between Cross-Sectional Area (CSA) ratio and vascularization grading system.

There was no significant linear association between neovascularization (considered as an ordinal variable) and clinical features or ultrasound measures, except for a positive linear association between the CSA ratio with the Ohberg score (rs: 0.453; *p* < 0.01, Figure 5): the greater the CSA, the higher the Ohberg score (the more tendon neovascularization) (Table 7).

## 4. Discussion

The results of this study found that non-elite sport players with painful unilateral tendinopathy exhibited: (1) greater patellar tendon thickness, width and CSA bilaterally when compared to a sample of asymptomatic non-elite sport players; (2) discriminative side-to-side ultrasound ratios suggesting asymmetry between the painful and non-painful knees; (3) significantly higher CSA ratio in non-elite sport players with higher tendon neovascularization; (4) significant association between CSA ratio with neovascularization and pain-related disability.

Current results suggest that non-elite sport players with painful unilateral patellar tendinopathy exhibit bilateral structural changes in the patellar tendon (i.e., higher CSA, width and thickness) compared to asymptomatic controls. Previous studies have observed higher thickness of the patellar tendon in elite sport player populations, both adults [13,15] or juniors [12,14]. However, the review conducted by Obst et al. found inconsistent evidence supporting the presence of imaging changes in the patellar tendon in elite sport players with patellar tendinopathy [16]. Our study is the first investigating the presence of gross ultrasound measures in a non-elite sport-player population with unilateral painful patellar tendinopathy. Current and previous findings support that painful patellar tendinopathy could be associated with ultrasound changes in both elite and non-elite sport player populations. This is an interesting finding since patellar tendinopathy has been commonly linked to elite/professional sport activities. However, it is important to note that we used clinical symptoms and imaging diagnosis of patellar tendinopathy in our sample of non-elite sport-players, which may limit the extrapolation of these results.

We found that 70% of non-elite sport players with painful patellar tendinopathy exhibited neovascularization in the tendon. Our results agree with those previously reported by Cook et al. who also observed neovascularization in 60% of painful patellar tendons in elite/professional jumping athletes [8]. These findings support the notion that pain is also present in the absence of neovascularization since at least 30% of non-elite sport players with patellar symptoms did not show vascularization. Preliminary evidence also suggests that the presence of neovascularization in abnormal patellar tendons can be associated with higher levels of pain and lower functional scores in elite sport players [8,24]. In non-elite sport players, we did not find such associations. Additionally, our results agree with those previously reported by Docking et al. [33]. These authors concluded that the extent of ultrasound imaging patellar tendon disorganization, e.g., hypoechogenicity or neovascularization, should not be used as a potential marker for the presence or severity of symptoms; however, once symptoms are present, structural changes in the tendon could promote pain [33]. It has recently been observed that the combination of two or more sonographic abnormalities can be related to more severe symptoms and may help explain clinical variations in elite sport players [34]. These findings agree with current assumptions that neovascularization in abnormal patellar tendons is not the only cause of symptoms. It is also possible that higher tendon loads suffered by elite sport players lead to greater tendon damage and pain symptoms, which would be less apparent in non-elite sport-players.

It has been suggested that the size of the hypoechoic tendon area may be linked to the amount of pain [11]. Our results support the notion that symptomatic patellar tendons have greater volume or size, as reflected by greater CSA, width and thickness; however, these morphological changes were not associated with clinical features. Additionally, we found that the asymptomatic patellar tendon of non-elite sport players with unilateral patellar tendinopathy also showed greater volume as compared to the control group. Our results also agree with previous data suggesting that imaging findings can be present in the absence of symptoms [35]. This is the reason why we assessed ultrasound ratios between symptomatic and asymptomatic patellar tendons in those subjects with pain. In fact, one novelty of this study was the analysis of ultrasound ratios for discriminating the symptomatic versus the asymptomatic patellar tendon. Although all the analyzed ultrasound ratios obtained excellent discriminative values, the best discriminate score was obtained by the width ratio suggesting that this ultrasound ratio measure could be better used as a guide for these clinical decisions. One potential explanation is that CSA and thickness ratios represent tendon volume measures and both maybe influenced by intra-tendinous vascular structures. Nevertheless, CSA and thickness ratios also had good discriminative values. In fact, CSA ratio was the only ultrasound measure associated with worse related-disability and higher tendon neovascularization supporting this hypothesis. Therefore, it is possible that the use of ultrasound ratios could have potential implications for implementation in clinical practice. For instance, identifying potential asymmetry in patellar tendon image measures, based on ultrasound ratios, may be a potential indicator of future injury in a non-elite sports player. Similarly, ultrasound ratios may also guide clinicians in applying therapeutic approaches with the aim to normalize this measure for avoiding side-to-side asymmetries in a particular individual, instead of reducing the CSA or thickness itself. Future studies should investigate clinical applications of ultrasound ratios for detecting tendon changes in different tendinopathies.

Finally, we should recognize the presence of potential limitations. First, the small number of non-elite sport players included could have led to unpowered results and overestimation of effect sizes. In fact, due to the small sample size and the non-probabilistic sampling method employed, this study should be considered as a pilot and current results should be confirmed or refuted in future studies. Second, all participants were men; therefore, current results should not be extrapolated to non-elite sports women. Third, all participants were active non-elite players, therefore we do not have data of non-active non-elite sport players. Similarly, we included individuals with painful patellar tendinopathy diagnosed with both clinical symptoms and ultrasound findings, e.g., presence of a hypoechoic area in the tendon. Since some patients with patellar tendinopathy might not exhibit hypoechoic areas in the patellar tendon, our results should not be extrapolated to the whole patellar tendinopathy population. Fourth, data about neovascularization should be considered with caution at this stage since the Ohberg score may not represent proper data on blood flow as compared to other outcomes, such as contrast-enhanced ultrasound blood flow. Future studies using more objective data will help to determine our results in terms of neovascularization. Fifth, the cross-sectional design of the study does not permit the determination of a cause-and-effect relationship between imaging findings and the development of patellar pain, particularly considering that non-elite sport players with unilateral painful patellar tendinopathy exhibited bilateral imaging changes. Longitudinal studies investigating ultrasound imaging changes during longer periods, e.g., one-year, could help to further elucidate the direction of our findings.

## 5. Conclusions

This pilot study found that non-elite sport players with painful unilateral patellar tendinopathy exhibit structural bilateral ultrasound changes and neovascularization within the patellar tendon when compared with asymptomatic non-elite sport players. Ultrasound ratios were able to discriminate between the symptomatic and asymptomatic knees in non-elite sport players. Current results suggest that ultrasound ratios could be a useful imaging outcome for identifying changes in the patellar tendon in sport players with unilateral patellar tendinopathy. Nevertheless, due to the small sample size and the non-probabilistic sampling method employed, current results should be further confirmed or refuted in future studies.

## Figures and Tables

**Table 1:** Anthropometry and clinical features of the sample. Values are presented as Mean (Standard Deviation) and *n*; %.

Clinical Features	Controls	Patients	*p*-Value
Age (years)	42.5 (5.5)	45.0 (4.5)	1.00
Weight (kg)	73.00 (8.1)	77.2 (9.6)	0.142
Height (cm)	176.5 (6.5)	169.8 (4.8)	0.001
BMI (kg/m^2^)	23.4 (2.0)	26.8 (3.2)	<0.001
NPRS (0–10 points)	-	5.2 (1.2)	
Time with pain (months)	-	4.9 (5.3)	
VISA-P (0–100 points)	-	42.1 (4.3)	
Medication (NSAIDs, *n*; %)	-	5 (25%)	
Ultrasound features			
Hypoechoic Area (*n*; %)	0; 0%	20; 100%	
Neovascularization (*n*; %)	0; 0%	14; 70%	
Vascularization grading system Grade 0 Grade I Grade II Grade III Grade IV	20; 100% - - - -	6; 30% 10; 50% 4; 20% - -	



BMI, Body Mass Index; NPRS, Numerical Pain Rate Scale; VISA-P, Victorian Institute of Sport Assessment-Patella; NSAIDs: Non steroid anti-inflammatory drugs.

**Table 2:** Spearman (95% confidence interval) correlation test between ultrasound gross measures and clinical features in non-elite sport players with unilateral patellar tendinopathy.

Ultrasound Measure	Time with Pain	NPRS	VISA-P
Thickness	−0.006 (−0.447 to 0.437, *p* = 0.980)	−0.103 (−0.521 to 0.355, *p* = 0.666)	−0.145 (−0.552 to 0.317, *p* = 0.543)
Width	0.074 (−0.380 to 0.500, *p* = 0.758)	0.026 (−0.421 to 0.463, *p* = 0.914)	0.009 (−0.435 to 0.449, *p* = 0.970)
CSA	−0.338 (−0.678 to 0.122, *p* = 0.145)	0.132 (−0.329 to 0.542, *p* = 0.580)	0.038 (−0.411 to 0.472, *p* = 0.873)

NPRS, Numerical Pain Rate Scale; VISA-P, Victorian Institute of Sport Assessment-Patella.

**Table 3:** Descriptive data (mean (SD)) and multiple comparisons for ultrasound measurements between patients with patellar tendinopathy and control subjects.

Ultrasound Measures	Controls (Mean Value)	Patients with Patellar Tendinopathy		Mean Difference (95% CI); Cohen’s d (a) Asymptomatic Knee vs. Healthy (b) Symptomatic Knee vs. Healthy (c) Symptomatic vs. Asymptomatic
		Asymptomatic Knee	Symptomatic Knee	
Thickness (cm)	1.01 (0.11)	1.55 (0.19)	3.22 (0.35)	(a) 0.54 (0.44 to 0.64); d = 3.48 † (b) 2.21 (2.04 to 2.38); d = 8.52 † (c) 1.67 (1.50 to 1.84); d = 4.68 †
Width (cm)	2.23 (0.44)	3.20 (0.40)	5.82 (0.45)	(a) 0.97 (0.70 to 1.24); d = 2.31 † (b) 3.59 (3.30 to 3.87); d = 8.07 † (c) 2.62 (2.42 to 2.81); d = 6.32 †
CSA (cm^2^)	2.07 (0.21)	3.08 (0.26)	5.43 (0.52)	(a) 1.01 (0.86 to 1.16); d = 4.27 † (b) 3.36 (3.10 to 3.61); d = 8.47 † (c) 2.35 (2.06 to 2.63); d = 3.85 †

CSA, Cross-Sectional Area; CI: confidence interval; † *p* < 0.001.

**Table 4:** Validity of ultrasound measurement ratios for discrimination between painful versus non-painful knees in non-elite sport players with unilateral patellar tendinopathy.

Stastistics	Ratios		
	Thickness	Width	CSA
ROC value (95% CI)	0.977 (0.941 to 1.000)	0.993 (0.974 to 1.000)	0.960 (0.907 to 1.000)
Youden Index	0.85	0.95	0.85
Cut-off Ratio Point	1.60	1.47	1.41
Sensitivity	95%	95%	100%
Specificity	90%	100%	85%
PPV	91%	100%	87%
NPV	95%	95%	100%
Positive LR	9.5	>95	6.7
Negative LR	0.06	0.05	0.00

CSA, Cross-Sectional Area; LR, Likelihood Ratio; NPV, Negative Predictive Value; PPV, Positive Predictive Value; ROC, Receiver Operating Characteristic.

**Table 5:** Spearman (95% confidence interval) correlation test between ultrasound ratios and clinical features in non-elite sport players with unilateral patellar tendinopathy.

Ultrasound Measure	Time with Pain	NPRS	VISA-P
Thickness Ratio	−0.149 (−0.554 to 0.314, *p* = 0.529)	−0.149 (−0.554 to 0.314, *p* = 0.529)	−0.184 (−0.579 to 0.281, *p* = 0.437)
Width Ratio	0.037 (−0.412 to 0.471, *p* = 0.878)	0.037 (−0.412 to 0.471, *p* = 0.878)	0.033 (−0.415 to 0.468, *p* = 0.892)
CSA Ratio	0.353 (−0.106 to 0.688, *p* = 0.126)	0.353 (−0.106 to 0.688, *p* = 0.126)	−0.581 (−0.815 to −0.195, *p* = 0.007) *

NPRS, Numerical Pain Rate Scale; VISA-P, Victorian Institute of Sport Assessment-Patella; CSA: Cross-Sectional Area; * Statistically significant (*p* < 0.017).

**Table 6:** Descriptive data and multiple comparisons for ultrasound measures according to the presence/absence of neovascularization.

	Vascularization Grading System Median (1st and 3rd Quartile); Mean (SD)		Mann–Whitney U Test *p*-Value
	Grade 0 (*n* = 6)	Grade I and II (*n* = 14)	
Clinical Outcomes			
NPRS (0–10)	4.5 (4.0–5.75);4.75 (1.0)	5.5 (4.0–6.0);5.31 (1.25)	*p* = 0.380
Time with pain (months)	9.5 (7.25–10.75);9.2 (2.6)	9.00 (8.0–11.75); 10.1 (2.8)	*p* = 0.737
VISA-P (0–100 points)	45.5 (41.25–46.75); 44.5 (3.1)	43.00 (37.25–45.75); 41.5 (4.5)	*p* = 0.184
Ultrasound Measures			
Thickness (cm)	3.25 (2.65–3.66); 3.2 (0.55)	3.15 (2.95–3.5); 3.2 (0.3)	*p* = 0.850
Width (cm)	5.8 (5.5–6.1); 5.7 (0.45)	5.9 (5.6–6.1); 5.85 (0.45)	*p* = 0.836
CSA (cm^2^)	5.0 (4.8–5.7); 5.15 (0.5)	5.55 (5.0–6.0.); 5.5 (0.5)	*p* = 0.276
Ratio Thickness (cm)	2.3 (2.05–2.7); 2.3 (0.4)	2.0 (1.8–2.4); 2.0 (0.35)	*p* = 0.148
Ratio Width (cm)	1.8 (1.6–2.0); 1.8 (0.2)	1.9 (1.6–2.2); 1.9 (0.3)	*p* = 0.591
Ratio CSA (cm^2^)	1.55 (1.5–1.65); 1.55 (0.1)	1.9 (1.7–2.0); 1.85 (0.2)	*p* = 0.017 *

CSA, Cross-Sectional Area; NPRS: Numerical Pain Rate Scale; VISA-P, Victorian Institute of Sport Assessment-Patella; SD: standard deviation; * Statistically significant.

**Table 7:** Spearman (95% confidence interval) correlation test between ultrasound gross and ratio measures and neovascularization (Ohberg score) in non-elite sport players with unilateral patellar tendinopathy.

Ultrasound Measure	Thickness	Width	CSA
Ohberg score	0.089 (−0.368 to 0.511, *p* = 0.710)	0.056 (−0.396 to 0.486, *p* = 0.815)	0.172 (−0.292 to 0.571, *p* = 0.469)
Ultrasound measure	Thickness Ratio	Width Ratio	CSA Ratio
Ohberg score	0.253 (−0.213 to 0.625, *p* = 0.282)	0.154 (−0.309 to 0.558, *p* = 0.516)	0.453 (0.014 to 0.745, *p* = 0.015) *

Statistically significant (*p* < 0.017).

## Supplementary Materials

The following are available online at https://www.mdpi.com/2075-4418/10/11/882/s1. Table S1. Validity of ultrasound measurement ratios for discrimination between patients with painful unilateral patellar tendinopathy and control subjects.
