# Supplementary material for: Ultrasound Characterization of Patellar Tendon in Non-Elite Sport Players with Painful Patellar Tendinopathy: Absolute Values or Relative Ratios? A Pilot Study
Source: Diagnostics (Basel). 2020 Oct 29;10(11):882. doi: 10.3390/diagnostics10110882 (PMC7694007; doi:10.3390/diagnostics10110882)
Supplement: Supplementary file 1 [file diagnostics-10-00882-s001.pdf]

Table S1. Validity of ultrasound measurement ratios for discrimination between patients with painful unilateral patellar tendinopathy and control subjects.

	**Ratios**		
	**Thickness**	**Width**	**CSA**
ROC value (95% CI)	1.000 (1.000 to 1.000)	1.000 (1.000 to 1.000)	1.000 (1.000 to 1.000)
Youden Index	1.00	1.00	1.00
Cut-off Point	1.82	4.14	3.55
Sensitivity	100%	100%	100%
Specificity	100%	100%	100%
PPV	100%	100%	100%
NPV	100%	100%	100%
Positive LR	>100	>100	>100
Negative LR	<0.001	<0.001	<0.001

CSA, Cross-Sectional Area; LR, Likelihood Ratio; NPV, Negative Predictive Value; PPV, Positive Predictive Value; ROC, Receiver Operating Characteristic.
